# Platinum nanoparticles: an exquisite tool to overcome radioresistance

**DOI:** 10.1186/s12645-017-0028-y

**Published:** 2017-07-11

**Authors:** Sha Li, Erika Porcel, Hynd Remita, Sergio Marco, Matthieu Réfrégiers, Murielle Dutertre, Fabrice Confalonieri, Sandrine Lacombe

**Affiliations:** 1 CNRS, UMR 8214, Institut des Sciences Moléculaires d’Orsay, Université Paris Sud, 91405 Orsay Cedex, France; 2 CNRS, UMR 8000, Laboratoire de Chimie Physique, Université Paris-Sud, 91405 Orsay Cedex, France; 3 Institut Curie/INSERM U759, Campus Universitaire d’Orsay, 91405 Orsay Cedex, France; 4 Synchrotron SOLEIL, BP48, Saint-Aubin, 91192 Gif-sur-Yvette, France; 5 CEA, CNRS, Institute for Integrative Biology of the Cell (I2BC), Univ. Paris-Sud, Université Paris Saclay, 91405 Orsay, France

**Keywords:** Metallic nanoparticles, Radioresistance, Radio-enhancement, Radiosensitization, *Deinococcus radiodurans*

## Abstract

**Backgroud:**

Small metallic nanoparticles are proposed as potential nanodrugs to optimize the performances of radiotherapy. This strategy, based on the enrichment of tumours with nanoparticles to amplify radiation effects in the tumour, aims at increasing the cytopathic effect in tumours while healthy tissue is preserved, an important challenge in radiotherapy. Another major cause of radiotherapy failure is the radioresistance of certain cancers. Surprisingly, the use of nanoparticles to overcome radioresistance has not, to the best of our knowledge, been extensively investigated. The mechanisms of radioresistance have been extensively studied using *Deinococcus radiodurans*, the most radioresistant organism ever reported, as a model.

**Methods:**

In this work, we investigated the impact of ultra-small platinum nanoparticles (1.7 nm) on this organism, including uptake, toxicity, and effects on radiation responses.

**Results:**

We showed that the nanoparticles penetrate *D. radiodurans* cells, despite the 150 nm cell wall thickness with a minimal inhibition concentration on the order of 4.8 mg L^−1^. We also found that the nanoparticles amplify gamma ray radiation effects by >40%.

**Conclusions:**

Finally, this study demonstrates the capacity of metallic nanoparticles to amplify radiation in radioresistant organisms, thus opening the perspective to use nanoparticles not only to improve tumour targeting but also to overcome radioresistance.

**Electronic supplementary material:**

The online version of this article (doi:10.1186/s12645-017-0028-y) contains supplementary material, which is available to authorized users.

## Background

Radiation therapies are used to treat many cancers. One of the major causes of radiotherapy failure and subsequent tumour relapse is the radioresistance of tumours to conventional treatments (Shu et al. 1998). The development of treatments to combat radioresistance is a major challenge. The understanding of mechanisms and pathways involved in radioresistance has motivated intensive studies on several model organisms, including *Deinococcus radiodurans,* a bacterium that can resist radiation exposure over 1000-fold greater than mammalian cells (Slade and Radman 2011). It has been shown that this organism exhibits an extraordinary ability to reassemble its functional genome after exposure to massive doses of radiation, while the genome of other organisms remains irreversibly shattered (Blasius et al. 2008; Confalonieri and Sommer 2011). Several groups have demonstrated that *D. radiodurans* resistance to radiation is attributed to a combination of physiological tools (Blasius et al. 2008; Levin-Zaidman et al. 2003; Daly et al. 2004), e.g. its efficient DNA repair machinery, its effective protection against oxidation of DNA repair proteins, and also the condensation of its nucleoid that may prevent dispersion of genomic DNA fragments produced by irradiation (Confalonieri and Sommer 2011). The resistance of *D. radiodurans* to radiation effects makes it an ideal candidate to probe the capacity of potential drugs such as NPs to enhance radiation effects in radioresistant cells and to characterize how these compounds may counteract the radioresistance mechanisms, and thus be subsequently explored in eukaryotic models.

For over a decade, nanomedicine has been proposed as a new strategy to improve radiotherapy treatments. Studies have been devoted to the development of tumour-targeting nanodrugs with the aim to improve the radiation effects in the tumour and diminish the exposure of healthy tissues to cytotoxic effects (Yhee et al. 2014; Kim et al. 2012; Escorcia et al. 2007; Hainfeld et al. 2010, 2013; Le Duc et al. 2011; Al Zaki et al. 2013). High-Z nanoagents, such as metallic (gold, platinum) and oxide (hafnium, gadolinium) nanoparticles (NPs), have been proposed as potential nanodrugs to amplify radiation effects. _ENREF_7 (Hainfeld et al. 2008; Porcel et al. 2010, 2014; Jang et al. 2011; Le Duc et al. 2014). In a pioneering study, Hainfeld et al. (2004) demonstrated that 1.9-nm gold NPs increase the effect of 250 kVp X-rays in the treatment of tumour-bearing mice. More recently, it has been shown that multimodal gold NPs improve not only the effect of ionizing radiation but also the performance of magnetic resonance imaging diagnosis (Miladi et al. 2014). Other metallic compounds, such as platinum complexes and platinum NPs (PtNPs), have shown excellent properties to amplify radiation effects (Usami et al. 2008; Charest et al. 2010; Porcel et al. 2012). Numerous studies, performed with various eukaryotic cells, have demonstrated the efficacy of high-Z NPs to enhance cell death in mammalian cells (Usami et al. 2008; Charest et al. 2010). This effect has been attributed to nanoscopic local dose deposition (Butterworth et al. 2012; Sancey et al. 2014). A relation between molecular damage and cell death has been established in the case of gadolinium NPs (Porcel et al. 2014). Surprisingly, the capacity of NPs to combat radioresistance in organisms treated by ionizing radiation has not yet, to the best of our knowledge, been reported.

Here, we report the effect of small PtNPs on *D. radiodurans*. In this perspective, we performed a toxicity study of PtNPs. The localization of PtNPs in *D. radiodurans* was characterized using two advanced microscopy techniques, namely Synchrotron Radiation Deep-UV fluorescence microscopy (SR-DUV) and high-angle annular dark-field scanning transmission electron microscopy (HAADF-STEM), which allows imaging of native NPs in bacteria without the use of any marker. The content of NPs in *D. radiodurans* cells was quantified by inductive coupled plasma mass spectrometry (ICP-MS). Lastly, we investigated the impact of NPs on the response of *D. radiodurans* to gamma-ray radiation exposure. This study opens the possibility to use small high-Z NPs to combat radioresistance.

## Methods

### Platinum NPs synthesis

Platinum NPs were synthesized by radiolysis as detailed elsewhere (Remita et al. 1996). Briefly, the PtNPs were produced from platinum salts Pt(NH_3_)_4_Cl_2_·H_2_O (Sigma-Aldrich**)** diluted in ultra-pure water (10^−3^ mol L^−1^) together with polyacrylic acid (Sigma-Aldrich) (0.1 mol L^−1^), and irradiated by 1.25 MeV gamma rays at a dose of 8 kGy with a dose rate of 33 Gy min^−1^. The platinum was thus reduced by solvated electrons and H· radicals induced by water radiolysis (Belloni et al. 1998) and aggregated to form PtNPs. Polyacrylic acid was used to coat the NPs and stop NP growth. UV–visible spectrophotometry was used to monitor the production of NPs. After irradiation, the peak characteristic of platinum complexes at 530 nm disappeared, which indicates the full reduction of platinum ions and thus production of PtNPs. TEM measurements were performed to characterize PtNPs size and shape. The NPs stored at 4 °C were stable for 3–4 weeks (Porcel et al. 2010). It is noteworthy to mention that the present synthesis method does not require any chemical compounds to reduce the metal. Moreover, after irradiation, the solution is sterile and ready-to-use, which is also a major advantage.

### Bacteria cultures

*Deinococcus radiodurans,* strain type R1, was inoculated onto solid TGY agar plates [0.5% bacto tryptone (Difco), 0.3% yeast extract (Difco), 0.1% glucose (Prolabo), 1.5% agar (Difco)] and grown for 3 days at 27 °C. A single colony was inoculated into 20 mL of TGY broth (0.5% bacto tryptone (Difco), 0.3% yeast extract (Difco), 0.1% glucose (Prolabo) and incubated 12 h at 27 °C in a shaker incubator. A volume of 0.5 mL of this exponentially growing culture was inoculated into 20 mL of TGY broth in a 100-mL flask. Bacterial growth was monitored by measuring the optical density at 600 nm (OD_600_).

### Toxicity of PtNPs

All of the experiments were repeated in triplicate on separate days. Bacteria were grown to early exponential phase (OD_600 nm_ ~ 0.3). Various volumes, ranging from 0 to 10 µL, of the PtNPs solution (10^−3^ mol L^−1^) were added to 100 µL aliquots of the bacteria medium containing approximately 10^7^ colony-forming units (CFU) of *D. radiodurans*/mL. The final ratios of PtNPs per bacterium added in the samples were equal to 0, 9 × 10^5^, 1.5 × 10^6^, 3 × 10^6^ or 6 × 10^6^ PtNPs per cell, which correspond to platinum concentrations of 0, 2.9, 4.8, 9.6 and 19.2 mg mL^−1^, respectively. These bio-colloids were incubated under agitation at 27 °C for 3 or 12 h. Solutions with bacteria free of NPs were used as controls.

### Bacteria growth

The impact of PtNPs on bacterial growth in liquid medium was performed as follows. Several 50 mL bacteria cultures were grown to early exponential phase (OD_600nm_ ~ 0.3). Various volumes of PtNPs were then added to the culture. The final ratio of PtNPs per bacteria was equal to 0, 0.9 × 10^5^ and 6 × 10^6^ PtNPs per cell, respectively. The suspensions were agitated in a shaker bath (Infors-HT Multitron) at 27 °C. Growth was monitored by measuring the optical density at 600 nm (OD_600_) at different time intervals.

### Cell irradiation

All of the experiments were repeated in triplicate on separate days. All of the experiments were repeated in triplicate on separate days. Before irradiation, the bacteria (with and without PtNPs) were centrifuged at 3000*g* for 15 min at 27 °C to remove cell media containing, or not, PtNPs. The pellet was re-suspended in fresh cell medium for radiation assays. Bacteria were irradiated by 1.25 MeV gamma rays (from a Cobalt 60 source) at increasing doses ranging from 0 to 8.0 kGy (dose rate of 1.4 kGy h^−1^).

The radiation assays were conducted on ice (4 °C) under atmospheric conditions, and the cell population remained constant during the irradiation procedure. There is no effect of these conditions on repair mechanisms of radioresistant prokaryotes, since cells are able to efficiently repair cell damage as soon as they are incubated at optimal growth temperature in fresh medium after irradiation (Bentchikou et al. 2007; Tapias et al. 2009). Non-irradiated samples (control cells) underwent all procedures except the irradiation step and were kept on ice. Immediately after irradiation, samples were analysed by clonogenic assay. Some irradiation assays were conducted in the presence of dimethyl sulfoxide (DMSO 1%), a well-known hydroxyl radical scavenger (Porcel et al. 2010).

The impact of NPs on cell survival after irradiation was quantified by colony-forming units (CFU) analysis. Briefly, serial dilutions of bacteria in TGY broth were prepared and plated on TGY agar plates. The number of colonies was counted after 72 h incubation at 27 °C. At least three independent experiments were performed for each irradiated condition and the errors were evaluated as standard deviations (SD).

### Statistical analysis

All curve fittings were performed with OriginLab^®^ software (Northampton, USA). The toxicity data of different PtNPs concentrations incubated for 3 or 12 h with *D. radiodurans* cells were analysed as a two-factor design (ANOVA: incubation time, concentration). The Turkey test was applied to compare the significant differences between conditions. The significance level was set at 5% and 1% (*p < 0.05, **p < 0.01).

### Synchrotron Deep-UV fluorescence microscopy

Synchrotron Deep-UV (SR-DUV) fluorescence microscopy, an alternative to confocal microscopy, was performed at the DISCO beamline at the synchrotron SOLEIL (Gif sur Yvette, France) (Kascakova et al. 2012). SR-DUV fluorescence microscopy has an excitation window down to 190 nm, which allows measuring the natural luminescence of NPs that absorb in the Deep-UV spectral range (below 350 nm). It thus prevents the use of fluorescent markers, such as Rhodamine, Cyanine-5 or BoDIPYs, which may influence the internalization and localization of NPs in the cells (Miladi et al. 2014). SR-DUV fluorescence microscopy has been previously used to follow the uptake of antibiotics in *Enterobacter aerogenes* and the internalization of gadolinium NPs in mammalian cells (Kascakova et al. 2012; Štefančíková et al. 2014).

In the present study, bacteria were centrifuged at 3000*g* for 15 min at 27 °C. The pellet was re-suspended in ultra-pure water and 0.5 µL aliquots of this cell suspension were immediately deposited on a quartz coverslip to perform SR-DUV microscopy.

Label-free PtNPs exhibited a maximum of emission at *λ*_em_ = 400 nm and a maximum of excitation at *λ*_exc_ = 290 nm (Additional file 1: Figure S1). Natural fluorophores, mainly NADH (*λ*_em_ = 460 nm, *λ*_exc_ = 340 nm), tyrosine (*λ*_em_ = 303 nm, *λ*_exc_ = 274 nm) and tryptophan (*λ*_em_ = 348 nm, *λ*_exc_ = 280 nm), generate an autofluorescence in bacteria (Štefančíková et al. 2014; Wagnieres et al. 1998; Kierdaszuk et al. 1995). To obtain the best contrast, SR-DUV images were thus recorded with an excitation wavelength of *λ*_exc_ = 298 nm and an emission wavelength of *λ*_em_ = 400 nm. Images were observed in bright field with a Zeiss Axioobserver Z-1. The objective was a 100× Zeiss ultrafar objective with glycerine immersion. The PtNPs fluorescence values were recorded with a dichroic mirror at 300 nm (OMEGA Optical, Inc., USA) and an emission band-pass filter QMAX/EM420-480 (OMEGA Optical, Inc., USA). The images were recorded with an acquisition time of 60 s using a CCD camera from Hamamatsu C9100-13 (HAMAMATSU PHOTONICS France SARL, France). The image analysis was performed with Image J (Rasband, W.S., ImageJ, U. S. National Institutes of Health, Bethesda, Maryland, USA, http://imagej.nih.gov/ij/, 1997–2011) software. The contribution of the intrinsic autofluorescence of bacteria was subtracted. The same contrast was chosen for all images.

### High-angle annular dark-field scanning transmission electron microscopy (HAADF-STEM)

HAADF-STEM was performed at the platform PICT-IBiSA (Institut Curie, Orsay France). This technique takes advantage of the high atomic number of platinum (*Z* = 78) compared with the organic elements H, C, N, O, P, S (*Z* < 16) (Nellist 1998; James and Browning 1999). HAADF-STEM images correspond to the electrons that cross the sample and are scattered at angles depending on the *Z*-numbers of the target atoms. Because the electrons are detected with an annular detector placed at variable height, the collection angle is set so that the contrast between elements of different *Z* is the maximum. The contrast of the HAADF-STEM signal is proportional to *Z* ^2^. Hence, the pixel intensity of light elements (close to 0) appears in grey in the images, while the maximum pixel value associated with the high *Z*-elements appears in white (Browning et al. 2012).

The biological samples consisted of 150-nm-thick slices of resin with embedded bacteria. The bacteria were centrifuged (Falcon(TM) type) at 3000*g* for 3 min at room temperature and fixed by mixing the suspension with a fixing buffer (glutaraldehyde 2%, paraformaldehyde 1%, phosphate buffer 0.1 M, pH = 7.4). The samples were then incubated 1 h at room temperature under 3D horizontal rotators (Orbitron Rotator, Boekel Scientific), and washed with phosphate buffer (pH = 7.4). To dehydrate the bacteria, the samples were embedded in 2% agar. Then, the pellets were washed in a series of ethanol baths (30, 50, 70 and 90%) and finally in *N*-(2-Hydroxypropyl) methacrylamide (HPMA)/ethanol mixed baths (HPMA/ethanol = 90:10, 95:5 and 97:3) for 10–20 min each. The pellets were incubated in HPMA/Epon resin (2:1, 1:1, 1:2) and finally in pure Epon resin bath for 3 h each. The pellets trapped in Epon resin were embedded in a silicon mould and incubated at 60 °C for 24 h to polymerize. Slices of 150 nm thicknesses were cut with an ultramicrotome. The ultrathin sections were deposited on carbon-formvar copper grids (Agar scientific).

The TEM experiments were performed with a Jeol 2200FS FEG electron microscope operating at 200 kV, using the 1-nm probe and a camera length of 6 cm. Statistical analyses of internalized particles were performed with ImageJ (Rasband, W.S., ImageJ, U. S. National Institutes of Health, Bethesda, Maryland, USA, http://imagej.nih.gov/ij/, 1997–2011). The Feret’s diameter (maximum calliper) was determined using a rolling-ball filter (50 px radius), after correction of the ramp effect and segmentation by manual threshold of the pixel intensity (Schneider et al. 2012).

### ICP-MS

*Deinococcus radiodurans* cells were cultured at 30 °C in 20 mL TGY 1× at O.D._600nm_ = 0.3. Seven aliquots of 1 mL were then incubated overnight with PtNPs (10^−3^ mol L^−1^) in the same culture conditions. Cells were filtered on a sterile nylon membrane (Millipore, 0.22 µm) and washed with 25 mL TGY broth to eliminate unincorporated NPs. Samples were stored at −20 °C. The quantification of platinum contained in cells, performed by ICP-MS, was performed by the UT2A Company (Pau, France). In parallel, a sterile membrane filter, washed with 5 mL TGY broth, as well as a solution of 7 mL *D. radiodurans* cell culture grown without PtNPs at the same cell density, was analysed as controls.

## Results and discussion

### Characterization of the platinum NPs size and toxicity

**Fig. 1:**
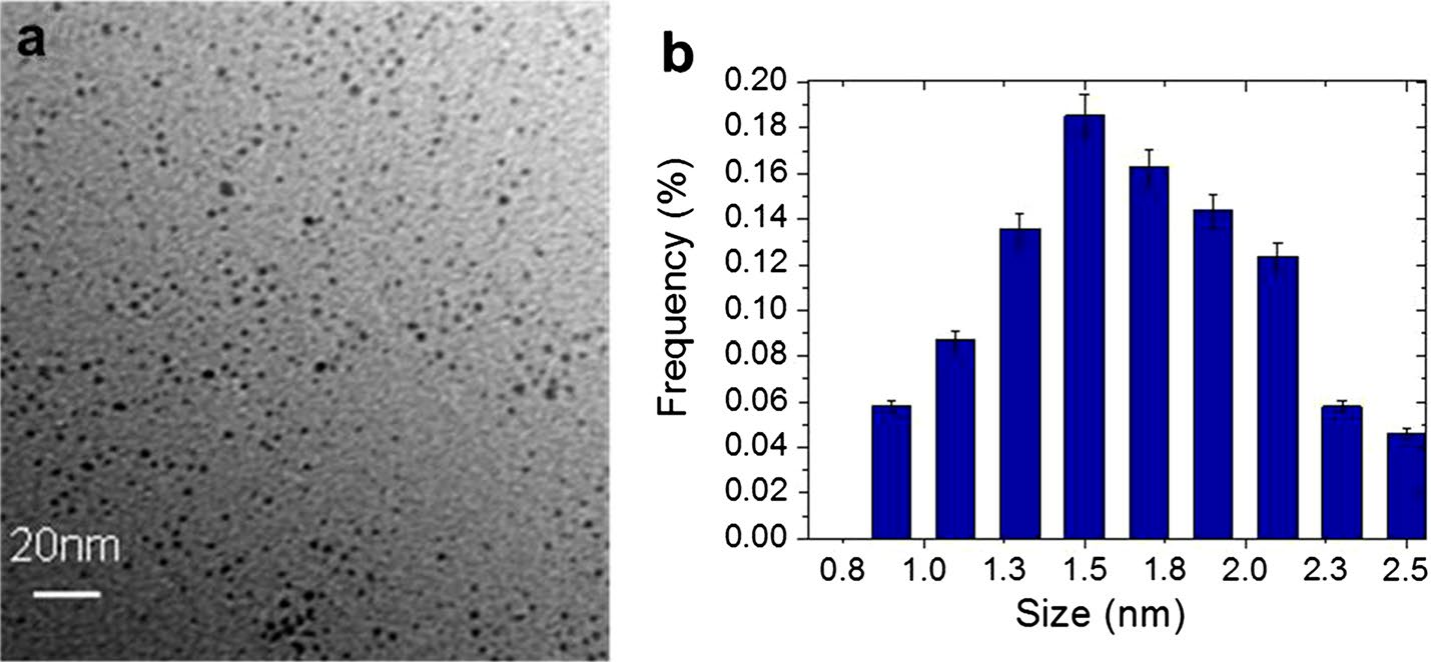
PtNPs characterization. **a** TEM image of PtNPs. *Scale bar* 20 nm. **b** Size distribution of PtNPs

TEM images of PtNPs are presented in Fig. 1. They show that PtNPs, synthesized by radiolysis, were spherical with an average diameter of 1.7 ± 0.8 nm.

**Fig. 2:**
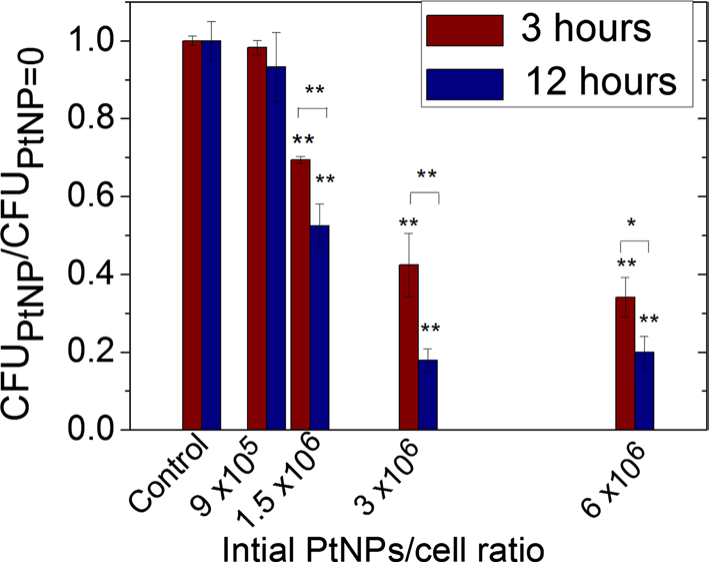
Number of CFU for *D. radiodurans* incubated with increasing NPs/cell ratios for 3 or 12 h at 27 °C (CFU_PtNP_), normalized to the number of CFU for the control (CFU_PtNP=0_) treated under the same conditions. Results were statistically analysed using a two-way ANOVA (Turkey test, *p < 0.05, **p < 0.01)

PtNPs toxicity was evaluated by comparing (i) the ability of an early log phase culture (OD_600_ = 0.3) of *D. radiodurans* to form colonies (CFU) and (ii) the bacteria growth parameters between bacteria loaded with PtNPs and bacteria free of NPs. These assays were performed with PtNPs concentrations ranging from 0 to 6 × 10^6^ PtNPs per cell. The cells were plated after 3 or 12 h of incubation with PtNPs. The number of CFU of *D. radiodurans,* incubated with PtNPs, over the number of CFU of *D. radiodurans* free of NPs (control) are reported in Fig. 2.

For the two incubation times, the CFU ratio decreased when the quantity of incubated PtNPs per cell increased, and was slightly more pronounced for the 12 h incubation. Indeed, at a concentration of 3 × 10^6^ NPs per cell, the CFU ratio dropped to 42% (±8%) after 3 h and to 18% (±3%) after 12 h. In both cases, the toxicity begins to be significant (>20%) at 1.5 × 10^6^ PtNPs per cell, which corresponds to a minimal inhibition concentration (MIC) of 4.8 mg L^−1^. This value is close to the MIC values obtained for other metallic NPs such as silver NPs (7.1 mg L^−1^) but lower than the values obtained for oxides NPs such as CuO and ZnO (200–500 mg L^−1^) (Bondarenko et al. 2013; Brayner 2008).

**Fig. 3:**
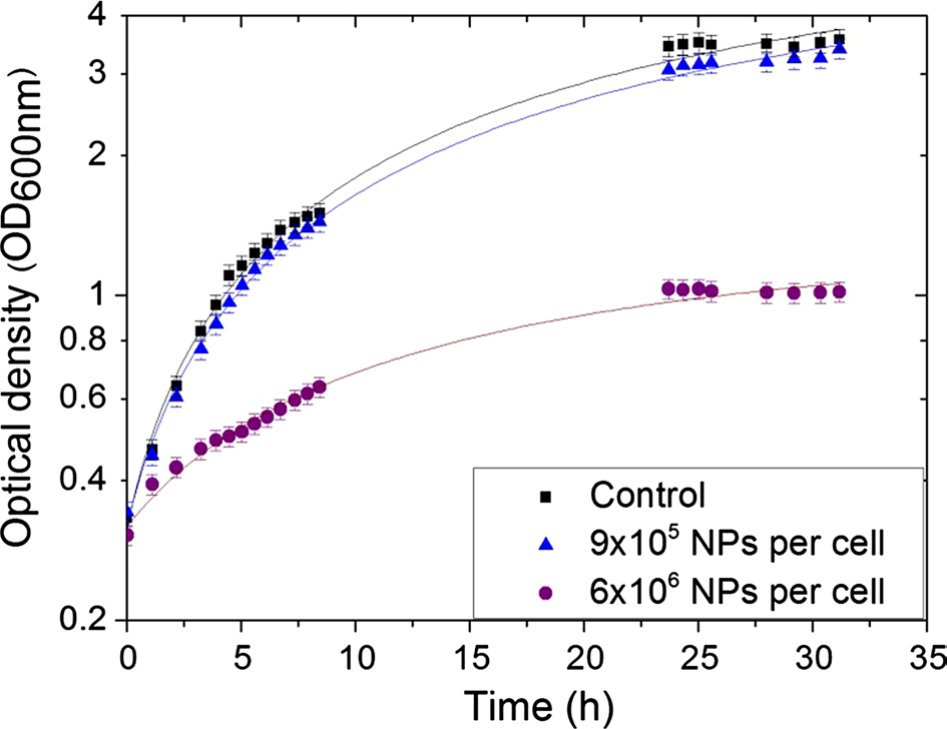
Growth curves of the control (*black squares*) and *D. radiodurans* incubated at 30 °C with NPs/cell ratios of 9 × 10^5^ (*blue triangles*) and 6 × 10^6^ (*purple dots*)

In addition, the impact of NPs on bacterial growth parameters at two PtNPs concentrations was investigated (Fig. 3). The addition to the cell medium of 9 × 10^5^ PtNPs per cell did not affect growth, whereas 6.0 × 10^6^ PtNPs per cell greatly impaired cell growth with a doubling time increase from 144 to 455 min.

For the following experiments presented here (microscopy and radiation experiments), we used a concentration of 9 × 10^5^ PtNPs per cell and an incubation time of 12 h, which corresponds to a toxicity of <5%, and an unchanged growth ability.

### Localization and quantification of NPs in *D. radiodurans*

#### SR-DUV fluorescence microscopy

**Fig. 4:**
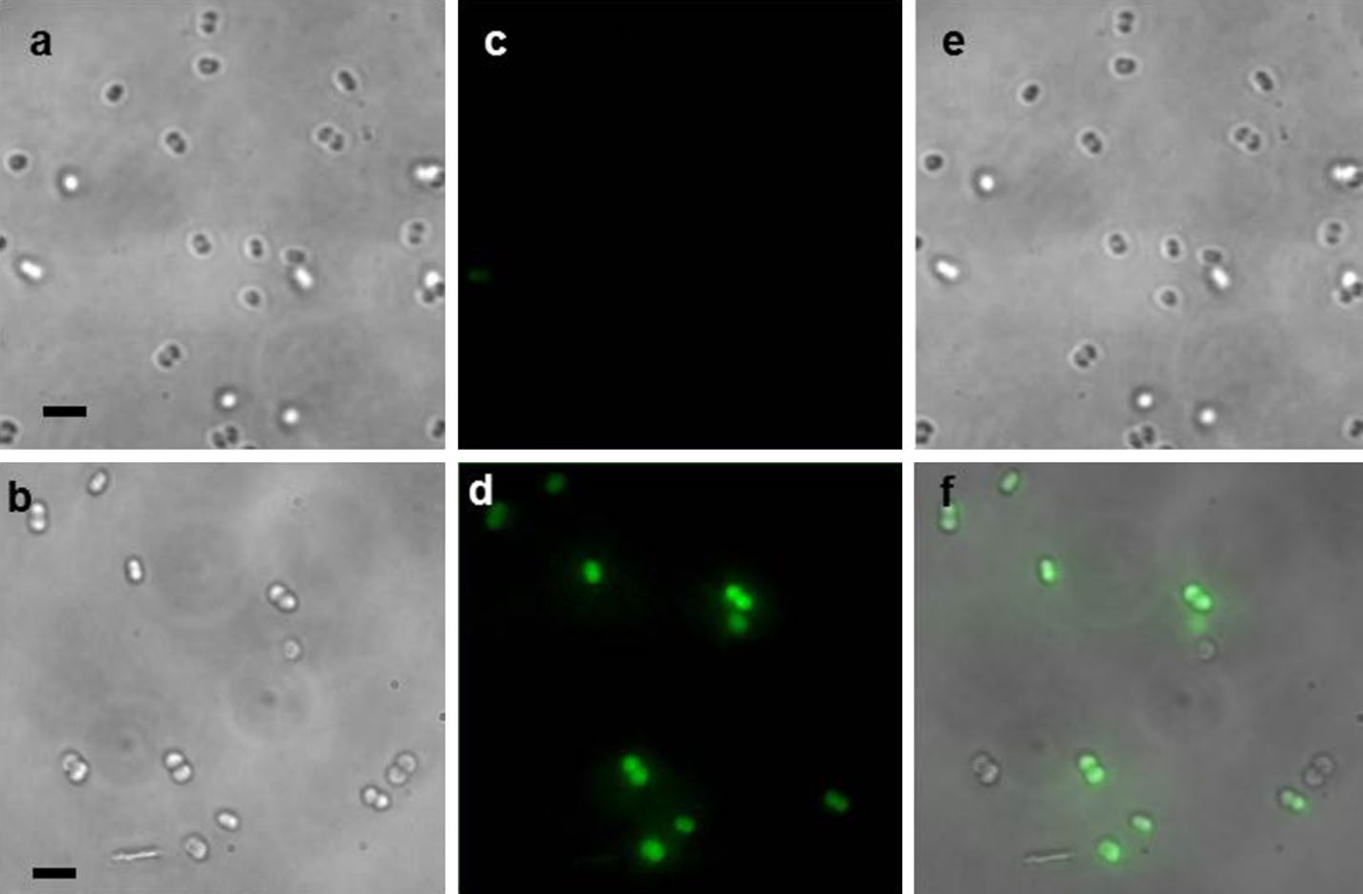
Light transmission images of **a** control cells and **b** *D. radiodurans* loaded with PtNPs during 12 h. Fluorescence images of **c** control cells and **d** *D. radiodurans cells* incubated with PtNPs for 12 h (the *green dots* correspond to the fluorescence signal of PtNPs). **e** Correspond to the merging of **a** and **c** images and **f** corresponds to the merging of **b** and **d** images. The *scale bar* is 5 μm

The transmission images of the control and PtNPs-loaded *D. radiodurans* cells (Fig. 4) show bacteria in their living state, which confirms that the cells were not extensively affected from the PtNPs incubation and the sample preparation. The good quality of the image demonstrates that SR-DUV microscopy is able to observe living cells without artefacts related to possible cell motion (only one cell shifted during the assay).

For SR-DUV fluorescence microscopy, fluorescent dots were only observed when PtNPs were pre-incubated with *D. radiodurans* cells. These green dots correspond to the intrinsic fluorescence emission of PtNPs (after subtraction of the autofluorescence). The merging of the transmission images showing the location of bacteria with the fluorescence images displaying the location of PtNPs confirmed that PtNPs were located within the *D. radiodurans* cells. As shown by the analysis of more than thirty images, no fluorescence was observed in the medium, and close to 80% of the bacteria contained internalized PtNPs.

#### HAADF-STEM

**Fig. 5:**
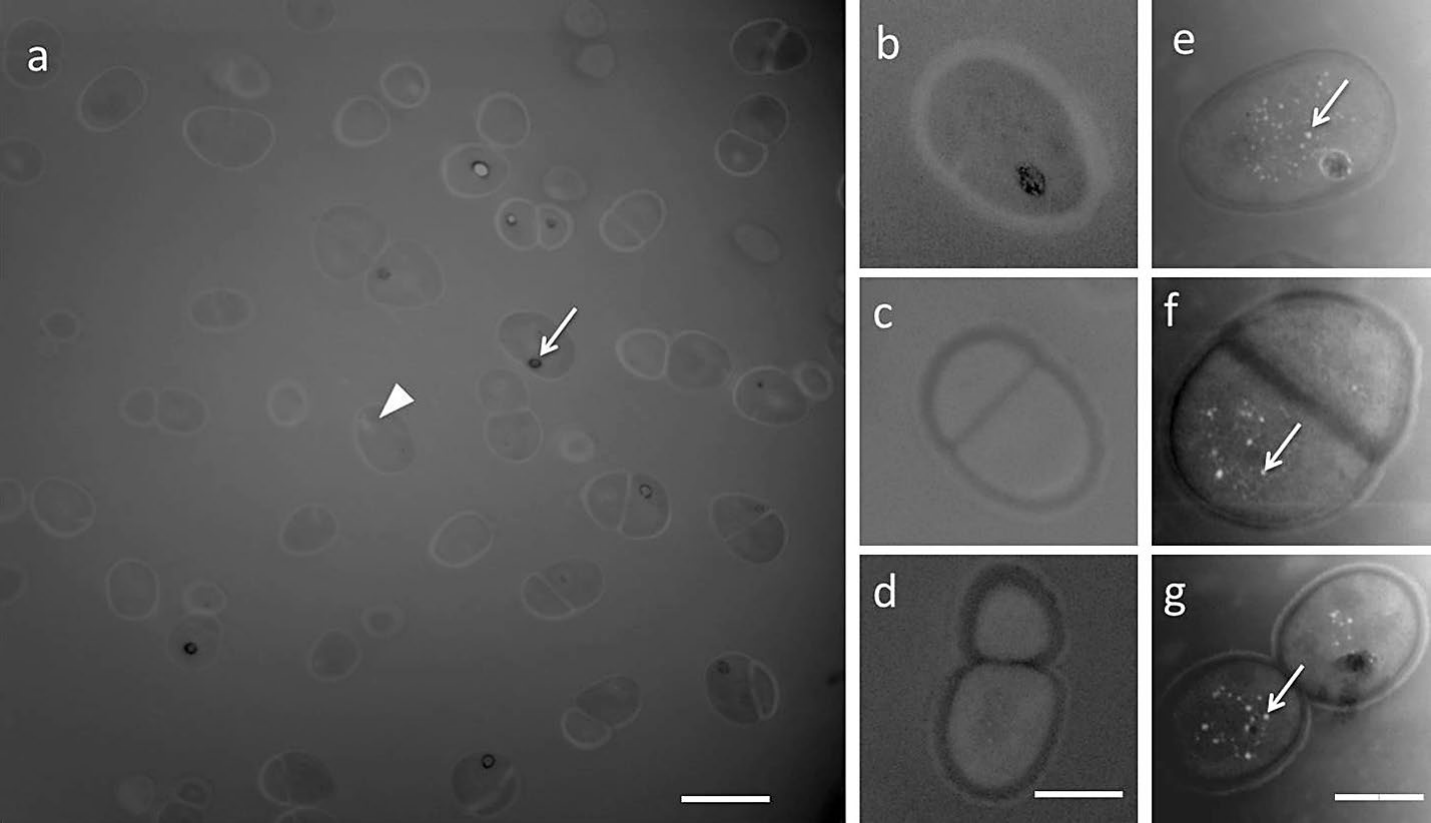
HAADF-STEM images. **a** Overall view of bacteria free of PtNPs (control). The *arrow points* to large electron-dense granules composed of polyphosphate and manganese and the *arrowhead* points to diffuse *light grey* regions associated with the nucleoid. The *scale bar* is 1 µm. **b**–**g** Representative images of the control (**b**–**d**) and cells incubated with PtNPs (**e**–**g**). *Arrows point* to small circular bright objects containing atoms with high atomic number that correspond to PtNPs. The *scale bars* are 0.5 µm

HAADF-STEM was used to increase the spatial resolution and distinguish the cytosol from the cell wall. Images of the control and PtNPs-loaded *D. radiodurans* are shown in Fig. 5. The well-known cell morphology of *D. radiodurans,* with the presence of single and diploid cells and the thick cell wall characteristic of gram+ bacteria, was clearly observable (Slade and Radman 2011; Levin-Zaidman et al. 2003; Eltsov and Dubochet 2005). A statistical counting, performed with >150 cells, demonstrated that ≈30% of bacteria (±3%, *p* = 0.95) contained large electron-dense granules in the cytosol (arrow in Fig. 5a). These granules are attributed to polyphosphate salts of manganese (Slade and Radman 2011). The diffuse light grey regions (arrow head in Fig. 5a) observed in the cytosol are associated with the nucleoid (Levin-Zaidman et al. 2003).

The observation of single and diploid cells of PtNPs-loaded *D. radiodurans* confirmed that PtNPs were not toxic and did not appear to perturb cell division. The cell walls (shape and thickness) of bacteria loaded with PtNPs did not show any noteworthy difference with control cells cultured without PtNPs. The bright objects observed in Fig. 5e–g correspond to PtNPs aggregated in the cytosol. Finally, these observations showed that PtNPs are present in the cytosol and not in the cell wall of *D. radiodurans*.

#### ICP-MS

A mass of 1.079 (±0.001) µg of platinum was obtained by the ICP-MS analysis of approximately 7 × 10^8^ *D. radiodurans* cells, which results from the overnight incubation at 30 °C of 7 × 10^7^ bacteria with ≈20 µg PtNPs and then rinsed several times prior to the measurement (see “Methods”). This mass corresponds to an uptake of 5% of the total amount of platinum. As expected, no platinum was detected in the controls, including the filter membrane and *D. radiodurans* cells grown without PtNPs, confirming that the detected platinum comes from the bacteria. In addition, the mass of platinum detected in the PtNPs solution (300 µL, 10^−3^ mol L^−1^ Pt) was 56.8 µg ± 0.1, which is close to the initial mass of Pt used for the preparation of the solution (58.5 µg ± 0.1). These experiments demonstrated that *D. radiodurans* cells internalized a mass of platinum of ≈0.154 × 10^−8^ µg (for a final population of 7 × 10^8^ bacteria), which corresponds to 0.0015 pg Pt per bacterium. PtNPs are composed of 1000 Pt atoms, with a mass of 3.25 10^−13^ µg each. Thus, the mass of 1.079 µg obtained by ICP-MS corresponds to 332 × 10^10^ PtNPs internalized in bacteria and an average number of 4700 PtNPs (=0.0015 pg Pt) per cell.

If we consider an average volume of ≈4.2 nm^3^ for a PtNP (2 nm diameter) and of ≈4.2 × 10^9^ nm^3^ for a *D. radiodurans* cell (diameter 2 µm), the volume occupied by 4700 NPs (1.97400 × 10^4^ nm^3^—for 7 × 10^8^ bacteria) corresponds to 0.0005% of the volume of each cell. This is in agreement with the STEM images, which shows isolated clusters of NPs sparsely distributed in the cytosol.

We compared our results with those observed for the internalization of NPs in eukaryotic cells. Studies with eukaryotic cells displayed masses of 0.3 pg for small gold NPs (5 nm) added to A431 epidermoid carcinoma cells after a 24 h incubation (Sha et al. 2016). Another study, performed with ~3-nm gadolinium NPs, showed an uptake of ≈0.6-pg gadolinium-based NPs in F98 glioma cells after a 5 h incubation (Taupin et al. 2015). Thus, *D. radiodurans* internalizes close to 200 times less metal than eukaryotic cells, on average. Interestingly, this value is comparable to the volume ratio of a 10-µm eukaryotic cell with a 1.5-µm bacterium, which is approximately 300. So an average bacterial cell internalizes NPs to the same extent as a eukaryotic cell.

### Influence of PtNPs on radiation effects

**Fig. 6:**
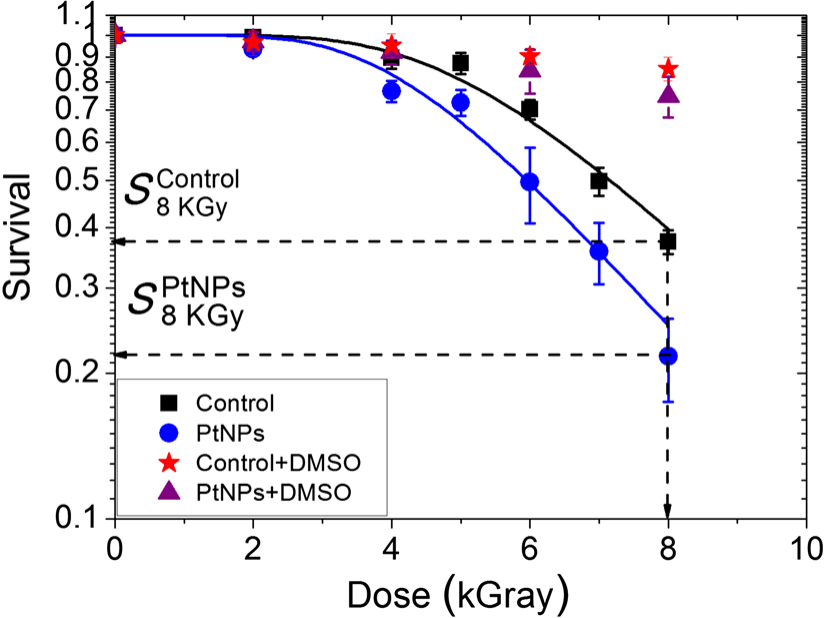
Survival of *D. radiodurans* cells after exposure at increasing doses of gamma-ray radiation. Control cells without DMSO (*black squares*), control cells with DMSO (*red stars*) *D. radiodurans* cells loaded with PtNPs (*blue circles*). *D. radiodurans* cells loaded with PtNPs and DMSO (*purple triangles*). $\varvec{S}_{{8\varvec{ kGy}}}^{{\varvec{Control}}}$ and $\varvec{S}_{{8\varvec{ kGy}}}^{{\varvec{PtNPs}}} \varvec{ }$ correspond to the survival at 8 kGy of the control and *D. radiodurans* loaded with PtNPs, respectively

The radiosensitizing effect of PtNPs in *D. radiodurans* was quantified by performing clonogenic assays with cells incubated with 9 × 10^5^ PtNPs per cell for 12 h before irradiation with gamma rays. The survival of *D. radiodurans* cells loaded with PtNPs with or without DMSO, and cells free of NPs (controls), were measured for radiation doses ranging from 0 to 8 kGy (Fig. 6).

Under our experimental conditions, PtNPs were not observed to be toxic to *D. radiodurans* and the plating efficiency of non-irradiated *D. radiodurans* and *D. radiodurans* pre-incubated with PtNPs was similar (data not shown). We observed that in bacteria free of NPs, the survival remained constant for doses up to 3.5 kGy and then decreased exponentially to 37% at 8 kGy. This result is in agreement with previous studies in which a cell survival close to 30% was observed at the same dose of gamma irradiation (Moseley and Mattingly 1971; Venkateswaran et al. 2000). In the presence of PtNPs, the dose at which the survival starts to decrease was shifted down to 2.7 kGy and the survival reached a value of 22% at 8 kGy. These effects may be quantified by two ways. First, the sensitization enhancement ratio (SER) at 50% is defined as the ratio of the doses associated to the same effect (50% CFU reduction) when cells are loaded, or not, with NPs. In the present experiments, the SER 50% is 1.17 (17% augmentation). On the other hand, this may also be quantified by comparing the number of CFUs obtained at the same irradiation dose for cells loaded, or not, with NPs. The amplification effect is 37% at 8 kGy. To the best of our knowledge, this is the first time that metallic NPs (4700 NPs; 0.0015 pg per cell) have been shown to significantly augment radiation effects (i.e. a decrease of cell survival) in the most radioresistant organism known.

In the presence of DMSO, the survival of *D. radiodurans* incubated in the absence or presence of PtNPs remained above 80–90%. This strongly implies that radiation-induced cell death and the amplification effect of PtNPs are likely driven by the production of hydroxyl radicals.

In this work, we attempted to use the model developed by Shuryak and Brenner to simulate the survival of *D. radiodurans* (Shuryak and Brenner 2009, 2010). This model focuses on the relation between proteins and DNA damage in the context of radiogenic oxidative stress. In spite of a greatly simplified representation of the complex biological processes involved, the authors succeeded to use this model to simulate the effect of radiation quality and low-dose effects on *D. radiodurans* survival (Shuryak and Brenner 2009, 2010). The simulation of the dose–response curves (*S*_cfu_) was used as follows.

1 $$S_{\text{cfu}} = 1 - \left( {1 - S} \right)^{4}$$

with

2 $$S = { \exp }\left( { - \alpha Dexp\left[ { - \beta \exp \left\{ { - \delta D} \right\}} \right]} \right).$$

In this model, *D* is the radiation dose (kGy), *α* (kGy^−1^) represents the induction of double-strand breaks (DSBs), *β* (dimensionless) corresponds to the capacity of the cell to repair DSBs and *δ* (kGy^−1^) represents the inactivation of protein activity by radiation. The parameter *β* is related to the cell culture conditions (e.g. growth medium composition, oxygenation) and to the intrinsic properties of the cells (e.g. genetic background, exponential or stationary phase of culture growth). The terms *α*, *β* and *δ* are interdependent. We first calculated *α* by expanding Eq. (2) at large *D* values.

For large *D* values (*D* ≫ 1), the terms $\left[ {{\mathbf{exp}}\left\{ { - \varvec{\delta D}} \right\}} \right]$ and $\left[ { -\varvec{\beta}{\mathbf{exp}}\{ - \varvec{\delta D}\} } \right]$ become small. Thus,

3 $${ \exp }\left[ { - \beta \exp \left\{ { - \delta D} \right\}} \right]\sim \left[ {1 - \beta \exp \left\{ { - \delta D} \right\}} \right].$$

Equation (2) becomes

4 $$S\sim {\text{exp}}( - \alpha D\left[ {1 - \beta \exp \left\{ { - \delta D} \right\}} \right]\sim { \exp }\left( { - \alpha D} \right).$$

*α* was calculated by fitting the survival curve at large doses with Eq. (4). *δ* was then calculated by fitting the total curve. The values of *α*, *β* and *δ* calculated for the control and for PtNPs-loaded *D. radiodurans* are displayed in Table 1.

**Table 1:** Parameters *α*, *β*, *δ* extracted from the simulation of the dose–response curves and corresponding Adjust R-Square (Adj *R* ^2^)

Samples	*α* [kGy^−1^]	*β* [dimensionless]	*δ* [kGy^−1^]	Adj. *R* ^2^
*D. radiodurans (control)*	0.290 ± 0.002	1.9 ± 0.3	0.35 ± 0.02	0.99
*D. radiodurans* + PtNPs	0.350 ± 0.005	1.9 ± 0.3	0.45 ± 0.04	0.98

We obtained an increase of *α* and *δ* with the addition of PtNPs. This suggests that the increase ienhancement effect is driven by the productionn cell death is due to the increase of non-reparable DNA damage (for ≈20% with *α* = 0.29–0.35) and to the inactivation of repair proteins (for ≈28% with *δ* = 0.35–0.45) likely due to oxidative stress. This is in agreement with the observation that the radio-enhancement effect is driven by the production of ⋅OH (Fig. 6).

As previously demonstrated, the size of the change induced by the activation of nanoparticles is of the order of few nanometers, i.e. the size of the nanoparticles (Porcel et al. 2010). This was shown in the case of carbon ions used as ionizing radiation (Porcel et al. 2010) and also with exposure to gamma rays (Additional file 1: Figure S2). Indeed, using pBR322 plasmids as nano-bioprobes to quantify nano-size damages, we observed that the induction of these alterations is amplified in the presence of nanoparticles (Additional file 1: Figure S2). As proposed by Porcel et al. (2010), we attribute the induction of these complex nano-damage events to the interaction of reactive nano-clusters composed of electrons and to ROS produced in the nano-volume around nanoparticles with the biomolecules. This confinement effect favors the production of complex damage events that may be more difficult to repair by the cells. This effect may also induce recombination of two ⋅OH in H_2_O_2_. One important feature of the radioresistance of *D. radiodurans* is the high capacity of bacteria to extrude H_2_O_2_ produced by ·OH dimerization, thus protecting them from oxidative stress (Daly et al. 2007); however, a local overproduction of H_2_O_2_ is expected to damage biomolecules and favour cell death. Thus, the presence of NPs is responsible for an enhancement of the relative biological efficiency of radiation exposure due to the spatial confinement of the dose effect (McMahon et al. 2011). This is in agreement with experiments performed with eukaryotic cells (Sha et al. 2016; Štefančíková et al. 2014; Porcel et al. 2014). An increase in lethal effects may be achieved if NPs are internalized and distributed in the vicinity of organelles (such as mitochondria, nucleus) of eukaryotic cells. Overall, the effectiveness of NPs in amplifying the effects of radiations is driven by physical chemistry factors, such as the capacity to produce ROS, including its localization and distribution within the cells.

## Conclusions

Using SR-DUV microscopy and HAADF-STEM to detect label-free nanoparticles, we demonstrated that ultra-small platinum NPs enter *D. radiodurans* cells in spite of its thick cell wall and that these nanoparticles have a MIC value of 4.8 mg L^−1^. We also showed that PtNPs, at a concentration of ≈4700 PtNPs per cell, do not have any major effects on bacterial growth under normal growth conditions. In spite of the high resistance of this organism to radiation, we found that this amount of PtNPs slightly but reproducibly increases cell death by 37% after exposure to gamma rays at a dose of 8 kGy. Our results also suggest that this amplification effect is due to the confined production of ROS in nano-volumes around nanoparticles, which favors the induction of complex damage in biomolecules. By simulation, we observed that this effect is likely able to impact the genome as well as the proteome of the bacteria. These early-stage nanoscale processes may affect the biomolecules of many other cell types including eukaryotic cells. Thus, this work opens up the possibility to use NPs to overcome the resistance of certain tumours to radiation, thus representing a potential major breakthrough in radiotherapy.

## Additional file

**Additional file 1.** Platinum nanoparticles: an exquisite tool to overcome radioresistance (supplementary data).

## Abbreviations

*D. radiodurans*: *Deinococcus radiodurans*
PtNPs: platinum NPs
SR-DUV: Synchrotron Radiation Deep UV
HAADF-STEM: high-angle annular dark-field scanning transmission electron microscopy
OD: optical density
DMSO: dimethyl sulfoxide
CFU: colony-forming units
SD: standard deviation
HPMA: *N*-(2-Hydroxypropyl) methacrylamide
TEM: transmission electron microscopy
MIC: minimal inhibition concentration
DSBs: double-strand breaks
