# Supplementary material for: Platinum nanoparticles: an exquisite tool to overcome radioresistance
Source: Cancer Nanotechnol. 2017 Jul 11;8(1):4. doi: 10.1186/s12645-017-0028-y (PMC5506239; doi:10.1186/s12645-017-0028-y)

Supplementary data for the manuscript entitled **Platinum nanoparticles: an exquisite tool to overcome radioresistance** by S. Li et al.

1. **Characterization of the PtNPs spectroscopic properties**

The fluorescence emission spectrum measured at the excitation of λ_exc_ = 290 nm and the fluorescence excitation spectrum measured at the emission of λ_em_ = 400 nm are presented in Figure S1. This measurement shows that label free PtNPs exhibit a maximum of emission at λ_em_ = 400 nm and a maximum of excitation at λ_exc_ = 290 nm

1. **Molecular scale impact of PtNPs activated by radiation**

The impact of nanoparticles on the induction of complex (nanosize) molecular damages was quantified by using pBr322 plasmids as a nano-bioprobes. This method, established elsewhere^15^ consists in the quantification of double strand breaks (DSBs), which are indicative of complex breaks larger than 2 nm (distance between two strands), as function of the irradiation dose. The effect of nanoparticles is investigated by adding nanoparticles in some samples. Briefly, pBR322 (Euromedex), a 4361 base pairs (2*.*83×10^6^ Da) plasmid, was diluted in TE buffer (10 mmol L^−1^ Tris-HCl pH = 7*.*6 and 1 mmol L^−1^ ethylenediaminetetraacetic acid (EDTA). Without irradiation, more than 95% of the plasmid molecules are under a supercoiled form and 5% under a relaxed form. The pBR322 plasmid was irradiated without NPs or with NPs at a ratio of 1 PtNP for 2 plasmids and with or without DMSO at doses ranging from 0 to 600 Gy. After irradiation, the samples were analysed by agarose gel electrophoresis.^[15]^ No significant artefact due to the presence of PtNPs was found during the electrophoresis, indicating that the addition of PtNPs did not damage the pBR322 plasmid. The dose response curves of nanosize breaks in plasmids free of nanoparticles (control) and in plasmids with PtNPs are presented in Figure S2. The yield of nanosize breaks given by the slope of the dose–response curve is close to 5.2 (±0.2)×10^-5^ plasmid^-1^ Gy^-1^ in the control and 7.8 (±0.3)×10^-5^ plasmid^-1^ Gy^-1^ in the presence of PtNPs. This corresponds to an amplification of 1.5 (±0.1). This analysis shows that the presence of PtNPs enhanced the yield of complex damages by 50%. The yield of complex breaks drops down to 0.7 (±0.1) × 10^-5^ plasmid^-1^ Gy^-1^ when DMSO is added to the samples. As previously described with carbon ions used as ionizing radiations ^[15, 16]^ the amplification of the radiation effects by nanoparticles is attributed to multi-scale processes. After activation by incident radiation, electronic rearrangements in the nanoparticles (Auger cascades, plasmon deexcitation…) result in the emission of electron bursts in the vicinity of nanoparticles. The interaction of these electrons with close water molecules leads to the production of reactive nano-clusters (HO**^.^** and byproducts such as H_2_O_2_). Thus, the increase of nano-size damage is attributed to the production of radicals confined in nano-clusters (mettre ici la reference Mc Mahon and co-workers).

**Figure legends**

**Figure S1**. Fluorescence excitation spectrum (λem = 400 nm) (purple dotted line) and fluorescence emission spectrum (λexc = 290 nm) (blue full line) of a label free PtNPs solution (10^-3^ mol L^-1^).


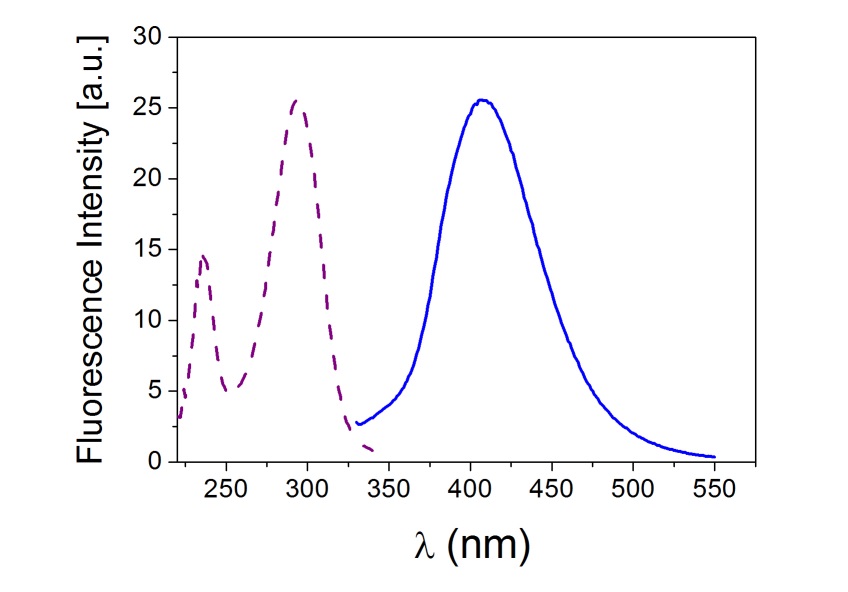


Figure S2. Average number of nanosize lesions induced in pBR322 plasmids, free of nanoparticles (black squares), in the presence of PtNPs (blue circles), in the presence of PtNPs and DMSO (purple triangles).


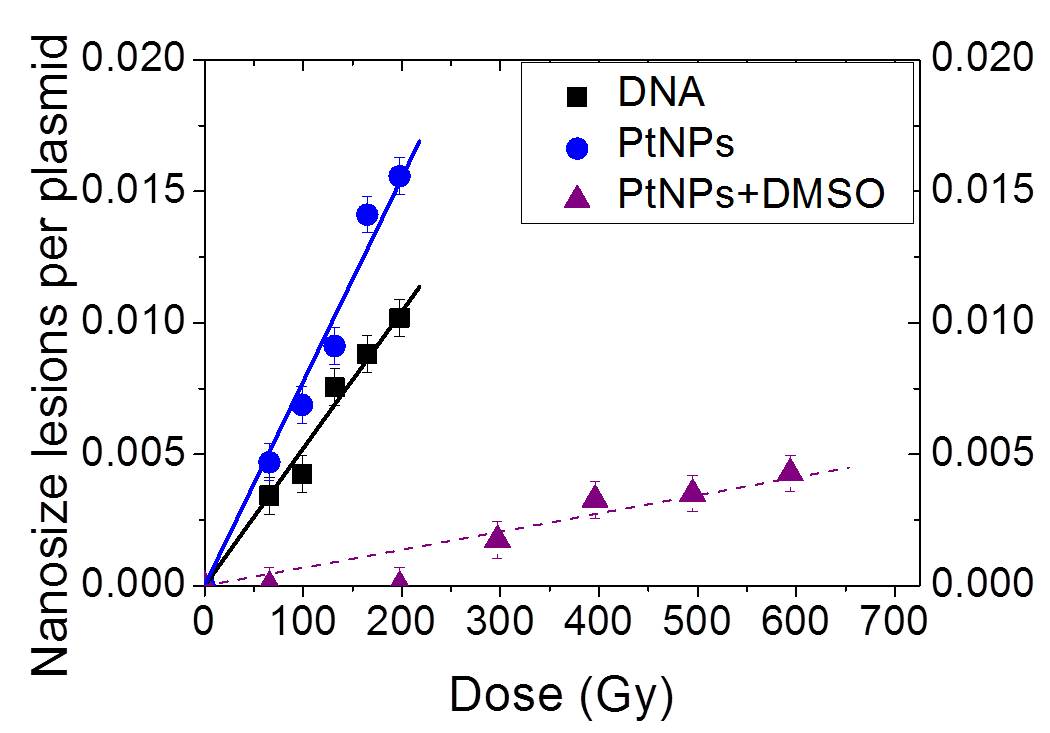

Supplement: Supplementary file 1 — Additional file 1. Platinum nanoparticles: an exquisite tool to overcome radioresistance (supplementary data). [file 12645_2017_28_MOESM1_ESM.docx]
